# Comparative Efficacy and Safety Study of Two Chondroitin Sulfate Preparations from Different Origin (Avian and Bovine) in Symptomatic Osteoarthritis of the Knee

**DOI:** 10.2174/1874312901307010001

**Published:** 2013-02-08

**Authors:** Patrice Fardellone, Mohammed Zaim, Anne-Sophie Saurel, Emmanuel Maheu

**Affiliations:** 1 Rheumatology Department, CHU Amiens Nord, INSERM ERI 12, Amiens, France; 2 Medical Department, Institut de Recherche Pierre Fabre, Toulouse France; 3 Project Department, Institut de Recherche Pierre Fabre, Toulouse France; 4 Rheumatology Department, Saint-Antoine Hospital, Paris, France

**Keywords:** Knee osteoarthritis, chondroitin sulfate, randomised clinical trial, noninferiority.

## Abstract

**Introduction:**

Some argued that clinical efficacy of Chondroitin Sulfate (CS) could vary upon the product origin. The objective of this trial is to compare the effect of 2 CS medicinal products from different origin: Structum^®^ (avian, 1000mg/day) and Chondrosulf^®^ (bovine, 1200mg/day).

**Methods:**

This was a randomized, double-blind, double placebo, active-controlled, parallel-group study using a non-inferiority design. Symptomatic osteoarthritis of the knee patients, according to American College of Rheumatology criteria, aged 50-80 years received either Structum^®^ (500mg BID) or Chondrosulf^®^ (400mg TID) during 24 weeks. Inclusion criteria were: global pain in the target knee ≥ 40mm on a Visual Analog Scale (VAS _0-100_), a Lequesne’s Algofunctional Index (LFI) score ≥ 7 (range: 0-24) and a radiological Kellgren-Lawrence grade 2 or 3. Primary outcome was the mean change over 24 weeks of pain VAS and LFI score. Secondary outcomes were patient’s and physician’s global assessments, Outcome Measures in Rheumatology Clinical Trials and Osteoarthritis Research Society International responders rate, analgesics intake and Medical Outcomes Survey Short-Form 12 (SF-12). Safety was assessed by recording adverse events. A non-inferiority test was performed on the Structum^®^-Chondrosulf^®^ difference for VAS and LFI score changes. Predefined non inferiority limit was settled as the lower limit of the 95% CI above -5mm and -1pt for pain VAS and LFI score respectively.

**Results:**

837 patients were randomized: 817 available for the full analysis dataset (FAS), 692 for the per protocol (PP) analysis. No statistical and clinical differences were observed for demographics and disease characteristics between the 2 groups. PP analysis showed no difference between groups on mean variations of pain VAS or LFI scores over 24 weeks. Mean Pain VAS decreased by 23.9mm (17.5) in Structum^®^ group and 23.8mm (17.2) in Chondrosulf^®^ group (difference: 0.012 [CI95%: -2.6 ; 2.6]). Mean LFI score decreased by 3.2 (2.4) and 3.1 (2.4) respectively (difference: 0.139 [CI95%: -0.2 ; 0.5]). The lower limits of the 2 CI were above predefined non inferiority margin, which demonstrated the non inferiority of Structum^®^ in comparison with Chondrosulf^®^. FAS analysis gave similar results. Secondary efficacy outcomes analysis showed the same trends. Responders rate were 76.3% and 73.8% respectively (PP, W24). Treatments were well tolerated: 2.4% in Structum^®^ group and 4.5% in Chondrosulf^®^ group withdrew from the study for safety reasons.

**Conclusion:**

Structum^®^ and Chondrosulf^®^ were equally effective in reducing functional impairment and relieving pain over 6 months in knee osteoarthritis patients, without any safety concerns.

**Trial Registration:**

http://www.controlled-trials.com Number: ISRCTN04305346.

## INTRODUCTION

Osteoarthritis (OA) is the most prevalent musculoskeletal disease and a leading cause of disability worldwide [1]. 40% of people over 70 years of age have osteoarthritis (OA) of the knee, with 25% of those with OA unable to perform daily activities of life and an additional 55% having some disability [2]. Radiographic OA of the knee may affect more than 37% of individuals aged 60 years old and over [3] and the prevalence of symptomatic knee OA in the United States has been estimated 12%. As a result of pain and functional impairment, it may severely alter the quality of life of patients [4].

Knee OA management is based on various therapeutic options which have been listed by several scientific international associations [5-7]. In general, treatment includes non pharmacological therapies such as physical therapy, exercise, patient education as well as pharmacological intervention. An acute flare of OA is usually treated with paracetamol, non-steroidal anti-inflammatory drugs (NSAIDs) or an intra-articular injection of corticosteroids for fast symptom relief. For non-acute treatment, symptomatic slow acting drugs for OA (SYSADOA) are available. Evidences supporting each of these options have been reviewed. Various international recommendations for the management of knee OA have been published in the recent past [5-7]. They all emphasize the importance of non-pharmacologic therapies such as education, muscle strengthening exercise, weight reduction, physiotherapy, spa-therapy [8] and physical aides such as crutches.

Chondroitin sulfate (CS) is one of the proposed SYSADOA. It consists of repeated chains of sulfated and/or unsulfated D-Glucuronic acid and N-acetyl-D-galactosamine residues. It is a major component of the cartilage matrix binding collagen fibrils and limiting water content by cooperating with hyaluronan. It plays a key role in the resistance and elasticity of the cartilage [9]. CS might be of various origins since its source is ubiquitarious. Current available medicinal preparations are extracted from avian, bovine, ovine or fish origins.

CS, either avian, bovine, ovine, porcine or fish, has been studied in human OA through many randomized clinical trials [10-20]. It has shown a clinical efficacy in reducing pain and improving functional status. It has also shown some properties in reducing the anatomic progression of the disease, by slowing down joint space narrowing. Clinical efficacy of CS has been summarized in several meta-analyses or systematic reviews which produced controversial conclusions [21-25], probably due to the variability of methodologies used and the level of efficacy observed in trials. Some have advocated that clinical efficacy of CS could vary upon the product origin, its quality, and its level of purity used in trials [26, 27].

Pharmacokinetic studies have shown that oral exogenous CS is absorbed as several metabolites [28], and the active moiety has not yet been identified. Furthermore, no optimal i.e. specific and sensitive assay method for use on biological fluids and determination of pharmacokinetics parameters has been yet developed. It is thus difficult to establish bioequivalence from plasma concentration against time curves.

Most of the trials have been conducted with pharmacological controlled preparations, namely Chondrosulf^®^ (bovine source ; IBSA Lab, Lugano, Switzerland) or Structum^®^ (avian source ; Pierre Fabre Labs, Castres, France) and have shown a superiority of both drugs over placebo on pain and function [10-18, 20]. These preparations fulfill the pharmacological quality controls during the fabrication process which ensures a high level of quality and purity of the products as well as a controlled dosage of the compound in each capsule. We therefore decided to design the present trial to demonstrate the non-inferiority of Structum^®^, one of the medicinal products available, versus Chondrosulf^®^ currently a widely used and studied medicinal CS indicated for the treatment of knee OA and marketed in several countries in Europe for many years, considered as the reference drug product. We also preferred to not include a placebo arm since it was deemed not feasible and somewhere unethical to conduct such a trial in a country where the 2 drugs were currently available and frequently used in daily ambulatory clinical practice by rheumatologists and general practitioners.

## PATIENTS AND METHODS

### Trial Design

This was a multicenter, prospective, randomized, double-blind, double-placebo, active-controlled, parallel group study performed in patients with symptomatic knee OA over a 24-week duration. The trial was designed as a non-inferiority trial [29].

### Patients

Patients were recruited by 126 centers in France, mostly general practitioners in primary health care settling. The study protocol and inform consent form received approval from the Ethical Board of Amiens before the study start. The trial was conducted in accordance to Good Clinical Practice and to the principles of the Declaration of Helsinki (1966, and its successive amendments).

To be included, patients having given their informed consent had to fulfill the following criteria: patients from either sex, aged 50-80 years, presenting with medial and/or lateral femoro-tibial OA of the knee according to American College of Rheumatology (ACR) criteria [30], symptomatic for more than 6 months, with a baseline level of symptoms as follows, global pain score on a Visual Analog Scale (VAS _0-100_) of at least 40 millimeters (mm) and a Lequesne’s algofunctional index (LFI _0-24_) score greater than or equal to 7 [31]. Patients had to show radiographic OA as defined by a Kellgren-Lawrence grade II or III [32] on an antero-posterior weight-bearing view of both knees taken during the 12 months prior to inclusion.

The main non-inclusion criteria were: secondary knee OA, ipsolateral painful hip OA, predominant patella-femoral disease, planned surgery of the target knee, treatment with systemic steroids during previous month, intra-articular corticosteroid injection during the 2 previous months, SYSADOA, bisphosphonates, strontium ranelate, knee lavage during the past 3 months or hyaluronic acid injections in the target knee during the past 6 months. Patients were also excluded if they took NSAIDS during the 2 days or paracetamol in the 12 hours prior to inclusion or had prior known allergy to the study treatment.

### Randomization

Patients were randomized to receive either Structum^®^ 500 mg bid or Chondrosulf^®^ 400 mg tid. The patients were assigned to one of the two groups according to a pre-established computer-generated global randomization list (treatment number) with balanced blocks of 4 treatments.

### Interventions

Both products were administered orally and daily during 24 weeks according to the following regimen Structum^®^ 500 mg bid (Avian CS, sodium salt, MA holder Pierre Fabre Médicament, Boulogne, France), and Chondrosulf^®^ 400 mg tid (Bovine CS, sodium salt, MA holder Laboratoires Genévrier, Sofia Antipolis, France), selected as the reference drug. Since the number of intakes differed from a product to another, a double dummy technique was used. Therefore each patient had to take 2 capsules three times a day. Placebo capsules were identical to each product, in order to allow for blinding. In accordance to European guidelines, a specific labeling was made for the trial.

In case of pain flare, a rescue medication was allowed, starting as recommended by all international guidelines [5-7] by paracetamol, allowed up to 4 grams per day for a maximum of 4 days. In case, paracetamol was ineffective to relieve pain, a NSAID could be used chosen in a pre-established list for which an equivalence score was available [33].

### Blinding Procedure

The study was double-blind, double-dummy. Both the patient and the investigator remained blinded throughout the entire study. All study case report forms recorded only the randomization number to identify the patient.

A decoding list was safeguarded at the sponsor's Clinical Pharmacy Department. The investigator and the pharmacist were each provided with a set of individual sealed decoding envelopes each corresponding to a treatment number.

An envelope could be opened only in case of absolute emergency.

### Outcome Measures and Clinical Assessments

Clinical assessments were performed at inclusion (V2), week 6 (V3), week 12 (V4), week 18 (V5) and week 24-end of study (V6).

#### Primary Outcome Measurements

In accordance with the European Agency for the Evaluation of Medicinal Products (EMEA) recommendations on clinical investigation of medicinal products used in the treatment of OA [34], pain relief and functional disability were assessed as the primary efficacy criteria in this study, using global pain score on a VAS for pain and the LFI score which ranges from 0 to 24 [31] for function. Global pain experienced during the last 24 hours prior to assessment was rated on a 100 mm VAS (0 = no pain, and 100, the most severe pain). The primary outcome measures were the comparisons between Structum^®^ and Chondrosulf^®^ groups of both the mean variation of the LFI and that of global pain over 24 weeks.

#### Secondary Outcome Measures

Secondary efficacy criteria included between-groups comparisons of the mean changes of pain scores (at rest or on motion rated on VAS) over 24 weeks, mean changes on patient’s and investigator’s global assessment scores at weeks 12 and 24 (VAS, where 0 is the worst and 100 the best assessment), mean changes of Physical Component Summary (PCS) and Mental Component Summary (MCS) of SF-12 (ranges 0-100) [35], and mean changes of each Osteoarthritis of the knee or hip Quality of Life dimension score (OAKHQOL) [36] between baseline and weeks 12 and 24, percentages of responders according to the modified Outcome Measures in Rheumatology Clinical Trials and Osteoarthritis Research Society International (OMERACT-OARSI criteria) [37] at weeks 12 and 24, and the consumption of analgesic medications (paracetamol and/or NSAIDs) over 24 weeks. These outcomes covered the core set proposed by the OMERACT for OA clinical trials [38].

Pain and LFI were recorded at baseline, week 6, 12, 18 and 24. Patient’s, investigator’s global assessments and quality of life were collected at baseline, week 12 and 24.

Safety was assessed at each visit by collecting adverse events spontaneously reported by the patient or identified by the investigator.

### Statistical Methods

Statistical analyses were conducted according to the statistical analysis plan initially approved by the Validation Committee of the trial.

Analyses were conducted on the following patient datasets: 1/ The Full Analysis Set (FAS): patients having received at least one administration of the product and having at least one evaluation of the primary criteria post administration. 2/ The "Per Protocol" (PP) dataset: subset of the FAS composed of all patients without any major protocol deviations. 3/ The Safety dataset: composed of all randomized patients having received one administration of the product has been used to perform the safety analysis.

The primary analysis was conducted on the PP dataset, as recommended by the EMEA guidelines for the conduct of non-inferiority trials [29].

The number and percentage of patients who withdrew from the study after randomization were provided by treatment group for all treated patients.

#### Handling of Missing Data

For the analysis of the primary criteria (global pain score and LFI) on both FAS and PP populations, the following rules of replacement of missing data were used: If the criterion was missing at one visit (due to either missing values or invalidation of the given visit), the mean between values on the visit preceding and the visit following the missing visit were used. If last visit was the missing visit, the value collected at week 18 has been carried forward. If two or more consecutive visits were missing, the same rules have been used (no patient is concerned with this later rule in the PP population). For the LFI, if one or several missing items prevented to calculate the score, no extrapolation of missing items was performed. For secondary criteria, only available assessments at each visit have been used.

Quantitative parameters have been described in each treatment group by means, standard deviations, minimum, median and maximum values. Qualitative parameters have been described in each treatment group by frequencies and percentages. Baseline comparisons of treatment groups characteristics have been performed in both the FAS and PP datasets. No formal statistical testing for homogeneity between the groups has been performed, as currently recommended in randomized trials.

#### Sample Size Calculation

For global pain score, the margin of non-inferiority has been set at 5mm, with an expected standard-deviation of 20 mm [17]. Six hundred and seventy-six patients (338 in each treatment group) were required to demonstrate non-inferiority of Structum^®^ with a Type I error rate of 2.5% and a power of 90% in the analysis on PP population. Assuming that 15% of patients would be excluded from the PP analysis, around 800 patients had to be included. For LFI, the margin of non-inferiority was set at 1. Based on previous data [17], the standard-deviation was expected to be 2.6. Two hundred and eighty-eight patients (144 in each treatment group) were required to demonstrate non-inferiority of Structum^®^ with a Type I error rate of 2.5% and a power of 90% in the analysis on PP population. Assuming that 15% of patients would be excluded from the PP analysis, around 340 patients had to be included. Thus, 800 patients were required to demonstrate non-inferiority on both criteria.

All analyses were performed using the SAS software (release 8.2).

#### Efficacy Analyses

Although analyses of the primary criteria were performed on the FAS and PP datasets, the analyses on the PP dataset were chosen as primary analyses, as recommended by current guidelines [29]. For both criteria, the change has been expressed as:

Change = Baseline-Mean (V3, V4, V5, V6) with V3 correspond to the week 6 visit, V4 to the week 12, V5 to the week 18 and V6 to the week 24 visit.

Therefore, a positive value means an improvement (decrease of pain or decrease of LFI).

The 95% confidence interval (95%CI) of the difference Structum^®^ *vs *Chondrosulf^®^ for both criteria has been calculated. Structum^®^ was to be declared non-inferior to Chondrosulf^®^ if the lower limit of the 95%CI was above -5mm for pain and -1 point for LFI both in the PP and the FAS populations.

No adjustment of the Type I error rate was required since each of the four analyses should demonstrate non-inferiority.

Analyses of the secondary criteria have been performed on the FAS and PP datasets. For all the secondary outcomes, the 95%CI of the difference between Structum^®^ and Chondrosulf^®^ has been calculated. These analyses were purely descriptive: no test of hypotheses (non-inferiority or superiority) has been performed.

For analgesics consumption, we analyzed the percentage of days of analgesics intake with respect to the theoretical number of days on treatment.

#### Safety Analyses

Any adverse event having been reported during the study for a given patient was classified by preferred term and corresponding system organ class using the the Medical Dictionary for Regulatory Activities (MedDRA version 7.1) terminology. Adverse events were classified as: treatment emergent adverse events, i.e., any adverse event occurring or worsening on study treatment during the randomized period, or non treatment emergent adverse events, i.e., any adverse event that occurs during the screening period or is reported as concomitant disease with the same intensity. Numbers and percentages of patients with at least one reported treatment emergent adverse event have been tabulated by treatment group.

## RESULTS

### Patients Disposition

**Fig. (1):**
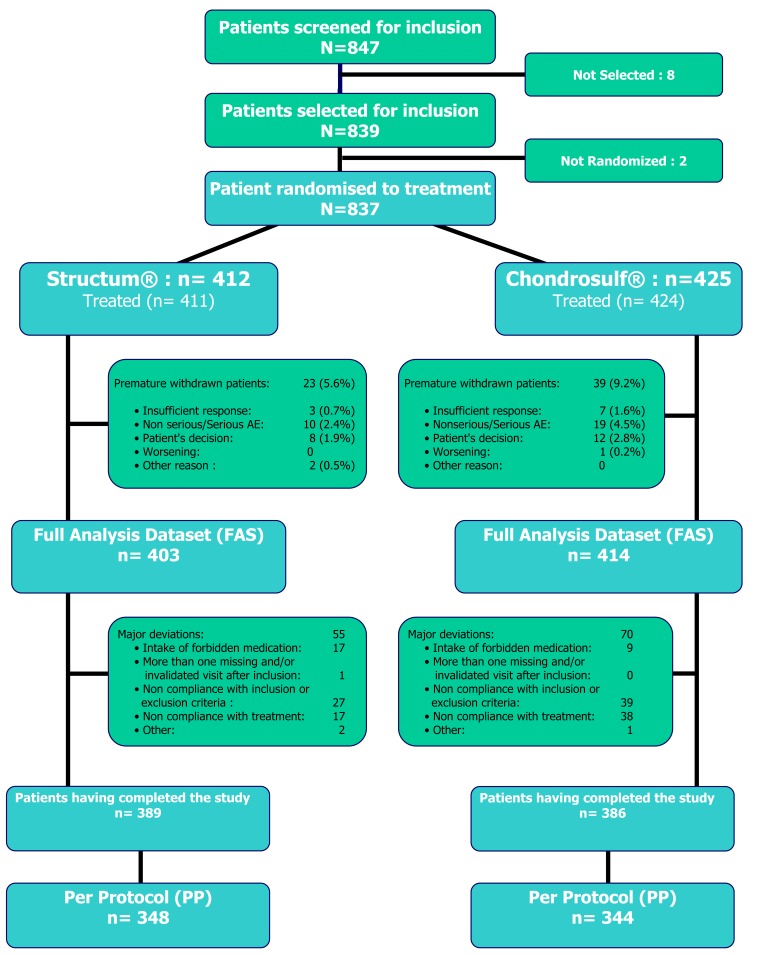
Disposition of patients in the trial.

Fig. (**1**) provides patient disposition throughout the study. A total of 847 patients were screened by 126 centres, 839 were selected, 837 randomized: 412 in the Structum^®^ group and 425 in the Chondrosulf^®^ group and 835 were effectively treated representing the FAS dataset. Sixty-two patients (7.4%) prematurely withdrew from the study (reasons in Fig. **1**) with a slightly higher proportion in the Chondrosulf^®^ group (9.2%). Major deviations were reported in 125 patients (15.3%), 55 in the Structum^®^ group and 70 in the Chondrosulf^®^ group. Most of these major deviations to the protocol were a/ a treatment duration lower than anticipated, b/ the intake of a forbidden medication and c/ a deviation to one of the selection criteria. All together premature drop-outs and major deviations left 692 patients, 348 in Structum^®^ group and 344 in Chondrosulf^®^ group in the PP dataset.

### Population Characteristics

Baseline characteristics in the PP population are given in Table **1**. Most of patients were women (68%), aged 65 years, with a mean BMI of 27.7, and a mean disease duration around 5.6 years. Almost 16% had a familial history of OA, around 40% had OA at another site (mostly spinal OA). Knee OA was bilateral in 75% of cases, at a radiographic Kellgren-Lawrence grade II for 70% and III for 30%.

Baseline clinical assessments of knee OA symptoms are presented in Table **2**. No differences between the treatment groups were observed. Baseline level of symptoms was high with a mean global pain on a VAS of around 62 mm, a mean LFI score of 11, and a mean SF-12 physical score of 37.

### Treatment Compliance

The mean overall compliance to treatment (estimated consumption / theoretical consumption x 100) reached almost 96% with no difference between groups and between FAS and PP population.

### Primary Efficacy Analysis

The results of the principal analysis (PP dataset) on main criteria (Mean variations of both global pain VAS and LFI score) are provided in Table **3**. Mean global pain VAS score over 24 weeks was 37.8 (17.0) and the mean change between baseline and mean over 24 weeks was 23.9 (17.5) in the Structum^®^ group. Corresponding figures in the Chondrosulf^®^ group were 38.5 (17.1) and 23.8 (17.2), respectively. The Structum^®^-Chondrosulf^®^ difference between mean changes on pain VAS was 0.012 with a 95%CI of [-2.58, 2.61]. The lower limit of the confidence interval (-2.58) was greater than the pre-defined non inferiority margin (-5.0) which allows concluding to the non-inferiority of Structum^®^ with Chondrosulf^®^ in the PP dataset for the global pain VAS score. Mean LFI score over 24 weeks was 7.8 (3.1) in the Structum^®^ group and 7.9 (3.1) in the Chondrosulf^®^ group. Mean changes (standard deviation) between baseline and mean index score over 24 weeks were 3.2 (2.4) and 3.1 (2.4) respectively. The between group difference for mean changes was 0.139 with a 95% confidence interval ranging from -0.22 to +0.50. The lower limit of the confidence interval was -0.22, greater than the pre-selected non-inferiority margin of -1.0, allowing concluding to the non-inferiority of the two products on the LFI score in the PP population. Since the non inferiority between Structum^®^ and Chondrosulf^®^ has been demonstrated for both co-primary criteria, it can be concluded that the trial demonstrated non-inferiority of Structum^®^ compared to Chondrosulf^®^ on both pain relief and functional improvement.

Analyses in the FAS dataset showed identical results on pain and function (Table **3B**).

### Secondary Efficacy Analyses

**Fig. (2):**
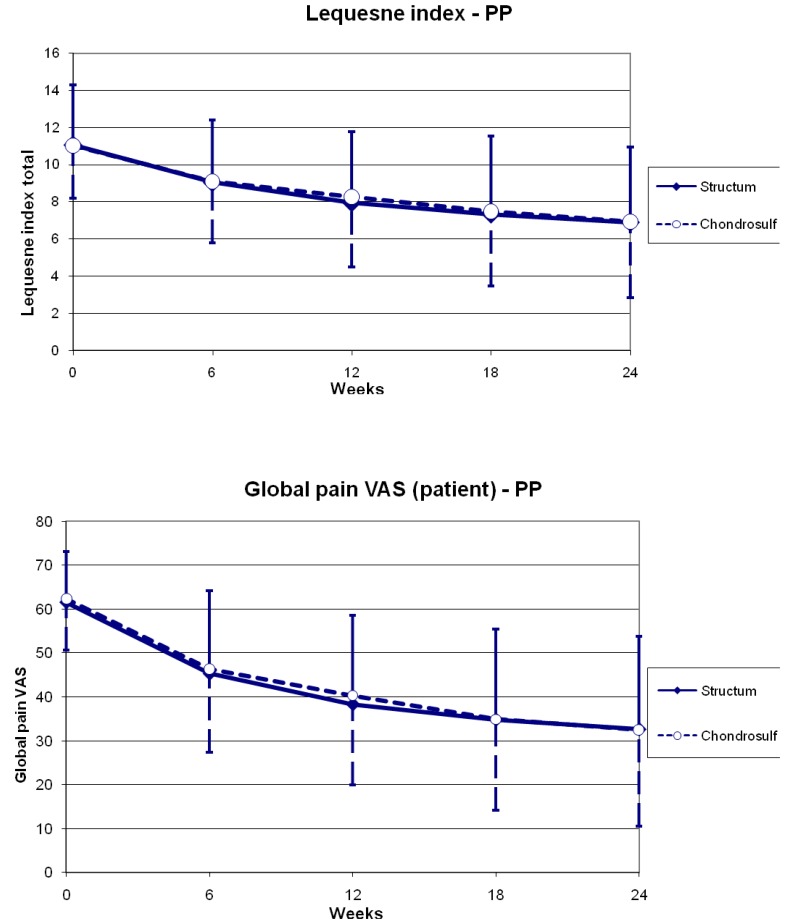
Evolution of Lequesne index score and global pain VAS between baseline and week 24 in the per protocol population.

In the PP population, the LFI score decreased by -4.1 (3.1) in the Structum^®^ group and -4.1 (3.2) in the Chondrosulf^®^ group, between baseline and week 24 (Fig. **2**). Global pain VAS score decreased by -29.0 mm (21.9) and -29.9 mm (22.3) respectively between baseline and week 24 (Fig. **2**). Such improvements are considered clinically significant.

All analyses of secondary outcome parameters (Table **4**) showed the same trends, without any intergroup difference at endpoint, whether in the PP or the FAS dataset. Pain on motion and pain at rest decreased the same way in both groups between baseline and week 24. Patient’s and investigator’s assessments showed a slight improvement in both groups. Slight but significant improvements of SF-12 Physical Component Summary and SF-12 Mental Component Summary were observed. Regarding OAKHQOL, there were also slight but significant improvements between baseline and week 24 for the physical activity, pain and mental health dimensions, but no significant improvement for social support, social activities and independent items (data not shown).

**Fig. (3):**
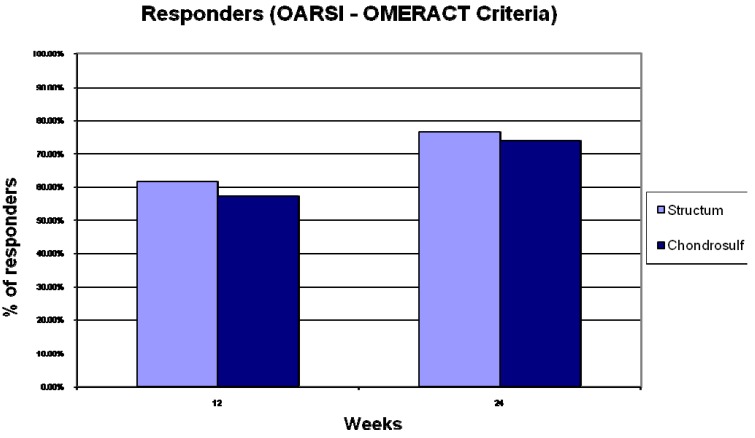
Responders rates according to the OARSI-OMERACT Criteria [PP].

Response rates according to the OMERACT-OARSI responder criteria at week 12 and 24 were high: 76.3% of responders in Structum^®^ group and 73.8% in Chondrosulf^®^ group at week 24, with no between group statistical difference (Fig. **3**).

In Structum^®^ group, 38.8% of patients and 33.1% in Chondrosulf^®^ group did not take rescue paracetamol medication during the trial. Among those who did, the majority took paracetamol on less than 10% of trial duration. No difference was observed between the two groups. Very few patients (<8%) took an NSAID during the trial (Table **4**).

### Safety Analysis

Table **5** summarises adverse events during the trial. Overall, 832 adverse events (AEs) were reported by 368 patients (44.1%), 392 by 177 patients (43.1%) in Structum^®^ group and 440 by 191 patients (45.0%) in the Chondrosulf^®^ group. A total of 392 treatment emergent adverse events (TEAEs) were reported by 177 patients (44.1%) and 438 TEAEs by 190 patients (44.8%) respectively.

Ten (2.4%) patients in Structum^®^ group and 19 (4.5%) in Chondrosulf^®^ group permanently discontinued the study drug and were prematurely withdrawn from the study due to an AE. The imbalance between the two groups with respect to premature discontinuation due to AEs is due to gastrointestinal disorders which occurred in 6 patients of Structum^®^ group (1.5%) versus 14 of Chondrosulf^®^ group 3,3%). Twenty serious adverse events (SAEs) were reported during the trial: five by 5 patients (1.2%) in Structum^®^ group and 15 by 14 patients (3.3%) in Chondrosulf^®^ group. None of these SAEs were considered being related to any of the treatments.

No relevant findings regarding physical examination were reported. No significant change with respect to vital signs occurred in any group between baseline and week 24.

Overall, treatment with both Structum^®^ and Chondrosulf^®^ were safe and well tolerated.

## DISCUSSION

This is the first report of a trial which compared the efficacy and safety of 2 CS of various origins (avian and bovine) and various daily dosages (1000 mg, 500mg BID, namely Structum^®^ and 1200 mg, 400mg TID, Chondrosulf^®^) on clinical symptoms in the treatment of knee OA. The results in the PP analysis clearly shown the non-inferiority of Structum^®^ compared to Chondrosulf^®^ both on functional impairment, assessed by the LFI score, and on global pain relief assessed by VAS, which were the co-primary outcomes. The same results were obtained in analyses on the FAS population. Accordingly, the same trends were observed on all secondary outcome parameters (patient’s global, investigator’s global disease assessment, quality of life, and responders rates using the OMERACT-OARSI response criteria).

This study is, to our knowledge, the first study who fully complied with the current European recommendations for the conduct of non-inferiority trials, in particular using the analysis performed on the PP population as main analysis, as recommended by these guidelines [29]. Moreover, this trial was in line with current European recommendations for the clinical investigation of medicinal products used in the treatment of OA [34], and their very recent revision (online January 2010) [39].

The first strength of our study, besides the true non-inferiority design and relevant statistical analysis, is the clear pre-trial definition of the non-inferiority margin allowing for concluding or not to non-inferiority. This was settled on the main co-primary outcomes, the LFI, as the lower limit of the 95%CI of the inter-group difference at endpoint above -1.0 and global pain score as the lower limit of the 95%CI of the inter-group difference at endpoint above -5 mm. According to Lequesne himself, the clinically relevant difference is around 1.5 to 2 points or more on the LFI score in knee or hip OA [31]. Most of publications in knee OA indicate that 9 to 10 mm on a pain VAS is the minimal clinically relevant difference, and therefore that the cut-off between a clinically and a non-clinically significant difference is 8 mm or below [40]. The between group differences observed in this trial are 0.139 [-0.22, 0.50] on the LFI score variation and 0.012 [-2.58, 2.61] on the pain VAS, which in both cases is close to zero. A second strength is the large size of the study population in which the trial has been conducted. Moreover, patients widely covered the spectrum of knee OA disease, since they were recruited in a primary health care setting. A third strength is the low number of patients “lost” between the number of randomized patients (safety dataset: n = 837) and the number of patients included in the PP dataset (n = 692), giving a difference of only 145 patients (17%), this difference being well balanced between the 2 groups. A fourth strength is given by the fact that statistical analyses performed on the FAS dataset fully confirmed the results observed in the PP analysis. In addition, all analyses performed on the secondary outcomes gave the same consistent results.

The absence of a placebo group could be considered as a limitation of this study. However, the reference drug Chondrosulf^®^ is a widely used CS indicated in the treatment of knee OA and marketed in several countries in Europe for many years. Its superiority over placebo has been reliably established and its safety well documented in several randomized, double-blind, controlled trials [11-15, 20]. In addition, participants and outcome measures in the present study were similar to those in previous published placebo-controlled trials with Chondrosulf^®^. Thus the use of Chondrosulf^®^ as active control seems fully justified according to the EMEA guideline “CPMP/ICH/364/96” [41].

All the arguments raised above justify the validity of the adopted study design i.e. a 2 arm study, head to head, to demonstrate the non-inferiority Structum^®^ versus Chondrosulf^®^ in term of efficacy according to the EMEA guideline “CPMP/EWP/2158/99” [29].

One other non inferiority trial of SYSADOA in knee OA which compared the efficacy on symptoms of avocado-soybean unsaponifiables with CS without placebo arm has recently been published providing results which are in line with our results comparing 2 CS medicinal products [42].

## CONCLUSION

In summary, this trial which was intended to compare 2 CS from different origin, Structum^®^ (avian, 1000mg/day) and Chondrosulf^®^ (bovine, 1200mg/day), has shown that both products are equally effective on pain relief and functional improvement in patients with symptomatic knee OA over a 6 month period of time. A clinically relevant improvement is obtained as early as week 6 and persists over the 24 weeks of the study. From this trial, it can be concluded that both products are clinically effective on symptoms reduction, and safe in the treatment of knee OA.

## Figures and Tables

**Table 1.** Patient Demographic and Baseline Characteristics in the PP Population

	Structum^®^ (N=348)	Chondrosulf^®^ (N=344)	All (N=692)
**Sex n (%) Female**	234 (67.2)	238 (69.2)	472 (68.2)
Age at screening (years)* Min., Max.]	65.15 (8.59) [49.00, 80.00]	65.09 (8.59) [50.00, 81.00]	65.12 (8.58) [49.00, 81.00]
**Weight (kg) **	75.71 (13.19)	75.23 (12.38)	75.47 (12.78)
**Height (cm)**	165.13 (8.30)	164.43 (8.77)	164.78 (8.54)
BMI (kg/m^2^;)* [1] Min., Max.]	27.67 (3.75) [19.00, 34.00]	27.74 (3.63) [19.00, 34.00]	27.71 (3.69) [19.00, 34.00]
**For females, post-menauposal status: yes n (%)**	230 (98.3)	235 (98.7)	465 (98.5)
**Disease duration (years)**	5.58 (5.37)	5.65 (5.30)	5.61 (5.33)
**Family history of OA n (%)**	51 (15.3)	54 (16.6)	105 (15.9)
**Target knee: right n (%)**	178 (51.1)	181 (52.6)	359 (51.9)
**Effusion in target knee: yes n (%)**	17 (4.9)	11 (3.2)	28 (4.0)
**OA in the non target knee: yes n (%)**	249 (71.6)	267 (77.6)	516 (74.6)
**Hip OA: yes n (%)**	22 (6.3)	22 (6.4)	44 (6.4)
**Cervical spine OA: yes n (%)**	71 (20.6)	55 (16.1)	126 (18.3)
**Lumbar spine OA: yes n (%)**	106 (30.6)	92 (26.9)	198 (28.8)
**Hand OA: yes n (%)**	35 (10.1)	17 (5.0)	52 (7.5)
**Kellgren-Lawrence grade n (%)** **Grade II** **Grade III**	244 (70.1) 104 (29.9)	238 (69.2) 106 (30.8)	482 (69.7) 210 (30.3)
**Non pharmacological treatment (target knee) n (%)**	13 (3.7)	13 (3.8)	26 (3.8)
**Previous IA corticosteroid injection in target knee: yes n (%) **	11 (3.2)	8 (2.3)	19 (2.7)
**Previous HA injections: yes n (%) **	15 (4.3)	11 (3.2)	26 (3.8)

All data are given as mean (sd) or N (%).

* Calculated data ; [1] BMI (kg/m²) = Weight (kg) / Height (m)²

OA = osteoarthritis ; IA = intra-articular ; HA = hyaluronic acid.

**Table 2.** Baseline Levels of Symptoms in the PP Population

Outcome Parameter Mean (SD)	Structum^®^ (N=348)	Chondrosulf^®^ (N = 344)	All (N = 692)
**Global pain VAS (0-100; mm)**	61.61 (11.62)	62.36 (11.69)	61.98 (11.65)
**Pain on motion VAS (0-100; mm)**	63.80 (14.80)	63.89 (14.73)	63.85 (14.75)
**Pain at rest VAS (0-100; mm)**	39.08 (20.81)	39.98 (21.21)	39.53 (21.00)
**LFI (0-24)**	11.05 (2.60)	11.03 (2.42)	11.04 (2.51)
**Patient’s global assessment of severity of the disease (mm)**	59.66 (17.52)	59.63 (16.74)	59.64 (17.12)
** Investigator’s global assessment of severity of the disease (mm)**	64.27 (16.24)	63.94 (16.50)	64.11 (16.36)
**SF-12 Physical (0-100) **	37.34 (7.77)	36.77 (8.03)	37.06 (7.90)
SF-12 Mental (0-100)*	47.12 (10.87)	47.00 (10.32)	47.06 (10.60)
OAKHQOL Physical activity (0-100) *	55.68 (18.17)	55.43 (18.01)	55.56 (18.08)
OAKHQOL Pain (0-100)*	51.08 (19.78)	51.31 (19.11)	51.19 (19.43)
OAKHQOL Mental Health (0-100)*	71.23 (20.38)	69.86 (20.83)	70.55 (20.61)
OAKHQOL Social support (0-100)*	57.43 (24.71)	58.26 (24.95)	57.84 (24.82)
OAKHQOL Social activities (0-100) *	66.08 (26.62)	67.20 (25.84)	66.64 (26.22)
**OAKHQOL Independent items (0-10) **	2.33 (2.16)	2.19 (2.13)	2.26 (2.14)

VAS = visual analog scale; mm = millimeters;

* Calculated data: VAS: 0 = very poor, 100 = very well (10 for OAKHQOL Independent items).

**Table 3A.** Results of the Primary Analysis (PP Dataset) on the Two Main Outcomes (LFI Score, Global Pain VAS)

Description	Statistic	Structum^®^ (N = 348)	Chondrosulf^®^ (N = 344)	Difference, [95% CI]

Global Pain VAS - Baseline (V2) *	Mean (SD)	61.61 (11.62)	62.36 (11.69)	
	[95% CI]	[60.38, 62.83]	[61.12, 63.60]	

Global Pain - Mean over 24 weeks *	Mean (SD)	37.76 (16.99)	38.53 (17.14)	
	[95% CI]	[35.97, 39.55]	[36.71, 40.35]	

Global Pain - Change (Baseline - Mean over 24 weeks *)	Mean (SD)	23.85 (17.54)	23.84 (17.24)	0.012 [-2.58, 2.61]
	[95% CI]	[22.00, 25.70]	[22.01, 25.66]	

LFI - Baseline (V2) *	Mean (SD)	11.05 (2.60)	11.03 (2.42)	
	[95% CI]	[10.77, 11.32]	[10.77, 11.28]	

LFI- Mean over 24 weeks *	Mean (SD)	7.82 (3.07)	7.94 (3.11)	
	[95% CI]	[7.49, 8.14]	[7.61, 8.27]	

LFI - Change (Baseline - Mean over 24 weeks *)	Mean (SD)	3.23 (2.42)	3.09 (2.43)	0.139 [-0.22, 0.50]
	[95% CI]	[2.97, 3.48]	[2.83, 3.35]	

V2 = visit 2 = baseline; 95% CI = 95% Confidence Interval;

* Calculated data.

**Table 3B.** Results in the FAS Dataset on the Two Main Outcomes (LFI Score, Global Pain VAS)

Description	Statistic	Structum^®^ (N=403)	Chondrosulf^®^ (N=414)	Difference, [95% CI]

Global Pain VAS - Baseline (V2) *	Mean (SD)	61.54 (11.68)	61.69 (12.27)	
	[95% CI]	[60.39, 62.68]	[60.51, 62.88]	

Global Pain - Mean over 24 weeks *	Mean (SD)	38.40 (17.21)	39.57 (18.05)	
	[95% CI]	[36.72, 40.09]	[37.83, 41.32]	

Global Pain - Change (Baseline - Mean over 24 weeks *)	Mean (SD)	23.14 (17.39)	22.12 (18.06)	1.017 [-1.42, 3.45]
	[95% CI]	[21.43, 24.84]	[20.37, 23.86]	

LFI - Baseline (V2) *	Mean (SD)	11.09 (2.58)	11.10 (2.44)	

	[95% CI]	[10.84, 11.34]	[10.87, 11.34]	

LFI - Mean over 24 weeks *	Mean (SD)	8.01 (3.15)	8.09 (3.24)	
	[95% CI]	[7.70, 8.32]	[7.77, 8.40]	

LFI - Change (Baseline - Mean over 24 weeks *)	Mean (SD)	3.08 (2.50)	3.02 (2.56)	0.067 [-0.28, 0.41]
	[95% CI]	[2.84, 3.33]	[2.77, 3.26]	

* Calculated data.

**Table 4.** Secondary Efficacy Outcome Measures Analyses (PP Dataset)

Outcome		Structum (N=348)	Chondrosulf (N = 344)	Difference [95% CI]
**Global pain (100 mm VAS)**	Change baseline - W24	-28.99 (21.94)	-29.91 (22.32)	
**Pain on motion (100 mm VAS)**	Change baseline - mean over 24 weeks	23.52 (18.00)	23.60 (18.78)	-0.086 [-2.83, 2.66]
**Pain at rest (100 mm VAS)**	Change baseline - mean over 24 Weeks	16.23 (20.45)	16.19 (18.89)	0.039 [-2.90, 2.98]
**LFI score**	Change baseline - W24	-4.13 (3.11)	-4.10 (3.17)	
**Patient’s global assessment**	Change baseline - W24	7.20 (21.11)	5.65 (20.60)	1.550 [-1.60, 4.70]
**Investigator’s global assessment**	Change baseline - W24	6.98 (18.72)	4.72 (18.19)	2.253 [-0.52, 5.03]
**SF-12-PCS**	Change baseline - W24	4.44 (8.67)	5.49 (8.12)	-1.053 [-2.40, 0.30]
**SF-12-MCS**	Change baseline - W24	1.66 (9.85)	1.57 (9.74)	0.099 [-1.48, 1.67]
**OAKHQOL-Physical**	Change baseline - W24	11.93 (18.12)	13.32 (16.68)	-1.393 [-4.02, 1.23]
**OAKHQOL-pain**	Change baseline - W24	16.71 (21.52)	17.23 (19.97)	-0.521 [-3.78, 2.74]
**OAKHQOL-Mental health**	Change baseline - W24	4.34 (16.46)	7.87 (15.09)	-3.528 [-5.92,-1.14]
**OARSI-OMERACT responder rate**	At week 24	76.3%	73.8%	
**Rescue medication paracetamol // NSAIDs**	% of patients who did NOT take rescue	38.8% // 92.2%	33.1% // 93.6%	

**Table 5.** Summary of AEs [Safety Dataset]

Safety Outcome	Structum (N=411)	Chondrosulf (N=424)
Number of AE	392	440
Number of TE AE	392	438
Number of Serious AE	5	15
Patients with at least one AE	177 (43.10)	191 (45.00)
Patients with at least one TE AE	177 (43.10)	190 (44.80)
Patients with at least one AE leading to definitive study drug discontinuation [1]	10 (2.40)	19 (4.50)
Patients with at least one AE leading to withdrawal [1]	10 (2.40)	19 (4.50)
Patients with at least one serious AE	5 (1.20)	14 (3.30)

[1] One patient discontinued the study follow-up on D47 due to an AE. He has not been discontinued from the study treatment.
